# Frequency of cytomegalovirus in fertile and infertile men, referring to Afzalipour Hospital IVF Research Center, Kerman, IRAN: A case-control study

**Published:** 2018-07

**Authors:** Majid Mohseni, Hamid Reza Mollaei, Seyed Alimohammad Arabzadeh, Tooraj Reza Mirshekari, Peyman Ghorbani

**Affiliations:** 1 *Department of Medical Microbiology, Kerman University of Medical Sciences, Kerman, Iran.*; 2 *Afzalipour In Vitro Fertilization Research Center, Kerman University of Medical Sciences, Kerman, Iran.*

**Keywords:** Infertility, Cytomegalovirus, In vitro fertilization, Real time PCR

## Abstract

**Background:**

Cytomegalovirus (CMV) virus can hide in urinary genital tract cells and affect male infertility disorders.

**Objective:**

To evaluate frequency of CMV in the semen samples of men with infertility problems referring to a in vitro fertilization (IVF) center in Kerman, Iran and its association with the parameters of semen.

**Materials and Methods:**

In this case-control study, Real time polymerase chain reaction test was performed for detection of human cytomegalovirus in 100 fertile men compared to 100 infertile men referred to the IVF center of Afzalipour Hospital, Kerman, Iran.

**Results:**

Out of 200 samples, 30 samples (15%) were positive for CMV DNA virus (23/100 men (23%) in case group and 7/100 men (7%) in the control group). Sperm counts and motility in the control group were more than the case group (p˂0.0001). There was a significant relationship between the prevalence of CMV infection and male infertility (p˂0.001).

**Conclusion:**

Our finding showed that, prevalence of CMV infection was higher in infertile men compared to fertile men and CMV infection can be considered as an important part of male infertility. So; antiviral treatment of positive cases can be effective in improving sperm quality and successful IVF. The relationship between CMV infection in semen and infertility was obtained in previous studies and was confirmed by our study.

## Introduction

Infertility is one of the most modern medical problems. Infertility is defined as a condition of reproductive system that pregnancy is not clinically acquired after 12 months of regular and unprotected sex (1). Approximately 15% of couples of reproductive age are defective in achieving fertility. In total, 40-50% of cases are related to males and in more than 50% of cases, the cause of male infertility remains unknown and is classified as infertility with unknown causes (2). 

Male infertility is often associated with genital infections which cause changes in inflammatory compounds of genital secretions (3). The cytomegalovirus (CMV) is a member of the herpes family, also called HHV5. Human cytomegalovirus (HCMV) or HHV5 causes asymptomatic infections in healthy individuals (4). Cytomegalovirus (CMV) can cause genital tract asymptomatic infections in men (5). The HCMV is endemic in some areas of the worldwide. Like all herpes viruses, CMV also has the ability to latent and re-activate and thus be able to cause a long-term infection (6). The mechanisms for regulating the latency of the virus is unknown (7). 

Many studies were done recently on CMV infection in people with immune deficiency; for example, in transplant recipients and HIV-positive patients but there is a little information about relationship between CMV and infertility (8). The prevalence of human cytomegalovirus DNA in reproductive organs and semen samples of fertilized and infertile men has been reported in a wide range of 8-65% (9). Given that we did not have accurate information on sexually transmitted infections in the geographical area of this study and there is no study about the frequency and relevance of cytomegalovirus infection and infertility in men, in this study frequency of CMV in semen samples of infertile and fertile men referred to an in vitro fertilization research (IVF) center located in Kerman, Iran. 

## Materials and methods

**Samples**

In this case-control study, semen samples of 200 men referred to the IVF Research Center of Afzalipour Hospital, Kerman, Iran from June 2016 to August 2017 were collected. For all participant’s complete semen analysis tests including sperm count, sperm motility, and morphology was performed. The case group were selected from infertile men referred to IVF center that had sperm motility<40% or sperm count< 15 million/ml, history of five years’ infertility, had no children and healthy partners with no identifiable cause of infertility. The men who had normal results of laboratory semen analysis according to the World Health Organization (WHO) standards, and sperm donors were selected for the control group(10).

**Semen analysis**

Samples were collected in a private room in the laboratory inside a sterilized container. Immediately the sample container was placed inside the incubator. The sample volume was measured and the macroscopic examination was performed 45 min after the sample collection. Samples were examined by microscopic examination with a wet glass slide after gentle shaking (10). In all cases, cellular elements, sperm motility (progressive, non-progressive, no movement), and sperm count were determined based on the 5th edition of the WHO (10).

**DNA extraction from semen samples**

DNA isolation of human cytomegalovirus from 200 μl of the sample was performed using a Viral DNA extraction kit according to the manual instructions (Roche, Germany). In the end, DNA was eluted in 50 µl elution buffer and was subjected to Nanodrop (Thermo Fisher, Germany) for quantification of DNA at 260 nm.

**Real-time polymerase chain reaction (PCR)**

All samples were tested for the presence of HCMV DNA by Real-time PCR method (Rotor Gene Q, Qiagen). Primers were synthesized against the highly conserved UL55 gene of HCMV to detect CMV viruses, and their sequences were as follows: 5′- TGG GCG AGG ACA ACG AA -3′ (sense); 5′- TGA GGC TGG GAA GCT GAC AT -3′ (antisense) and specific probe was FAM- TGG GCA ACC ACC GCA CTG AGG -BHQ1; which were designed using Primer3 plus online tools (www.bioinformatics.nl/cgi-bin/primer3plus/ primer3plus.cgi). For Real-time PCR test, 10 µL DNA was added to 10 µL reaction mixture preparing from TaqMan Probe Master Mix (Ampliqon, Denmark).

 Reaction mix containing Taq polymerase enzyme, gelatin, 0.6 µmol/L of each primer, 0.2 µmol/L probe, deoxynucleotid triphosphate mix, and reaction buffer (KCl, L Tris-HCL, Mg-Cl2 pH=8.3). PCR was done at 50^o^C, 2 min for activation of UNG and at 95^o^C for 15 min for activation of Taq Polymerase and first denaturation, followed by 45 cycles of 95^o^C for 15 secs, 60^o^C for 40 secs and in the 60^o^C fluorescence was detected in green channel (FAM) for specific product and yellow channel (HEX) for Internal control. DNA extracted from standard positive sample KSG1 (10^4^ copies/ml) and KGS2 (10^2^ copies/ml) (Interlabservice, Russia) were included in the run as positive controls, while a mixture without DNA template was used for negative controls.

**Ethical consideration**

This study was approved by the Ethics Committee of Kerman University of Medical Sciences,Kerman,Iran (IR.KMU.REC1395.97). Written informed consents were obtained from all participants before enrollment.

**Statistical analysis**

All statistical analyses were performed using SPSS statistical software (Statistical Package for the Social Sciences, version 18.0, SPSS Inc., Chicago, IL, USA). Comparison of the mean sperm count, motility, and morphology between CMV positive samples and negative samples was performed with the independent Student’s *t*-test, independent sample T test, Kolmogorov-Smirnov and Mann–Whitney test used to assess differences. A P. Value less than 0.05 was considered as statistically significant. 

## Results

200 semen samples were analyzed from men referred to the IVF Research Center of Afzalipour Hospital, Kerman, Iran. The Mean±SD of participant’s age was 34.5±4.86 yr old (34.84±5.03 yr in the case group and 34.24±4.69 yr in the controls). Demographic and sperm analysis variables are shown in table I.

**Table I:** Distribution of parameters in two study groups

**Groups**		**N**	**Age**	**Volume**	**Count**	**Motility**	**Morphology**	**Round cell**
Case group								
	Oligo spermia	39	35.51± 4.39	3.38± 2.12	6.64 ±4.52	28.03± 21.21	6.82 ±3.26	4.74±6.58
	Azoo spermia	19	34.10± 5.9	2.35± 1.6	0	0	0	1.21±2.87
	Motile less than 40%	42	35.54± 5.16	3.02± 1.70	75.7 ±42.76	26.30± 17.14	8.71 ±3.27	4.02±3.69
Control group		100	34.24 ±4.69	3.32± 1.68	90.98 ±31.45	65.7± 4.54	8.9 ±3.26	3.59±4.60
Total samples		200	34.5±4.86	3.18±1.7	62.6±47.9	26.5±4.2	7.6±4.03	3.68±4.82

Data presented as Mean±SD. We describe the data by mean and standard deviation tests, Median test and ratio test

In the case group (n=100), there were 39 oligospermia samples (19.5%) with the sperm count<15 million/ ml and 19 azoospermia samples (9.5%) without sperm. In 42 samples (21%) sperm motility was less than 40%. Real time PCR was done for all of 200 semen samples. From the total of 39 specimens in the case group with oligospermia, 7 samples (17.9%) were positive for CMV DNA. The prevalence of the CMV infection in oligospermia cases was obtained 17.9% and 5.3% in azoospermia specimens.

Also, from 42 samples with less than 40% motility, 15 (35.7%) cases were positive for CMV DNA (Table II). In the infertile group, 23 samples were positive for CMV DNA and there was a significant correlation between the causes of infertility (oligospermia, azoospermia, and motility less than 40%) and CMV DNA (p=0.01). According to the analysis of the results based on statistical analysis among the studied groups, it was determined that the parameters of count, motility, and morphology have a direct and significant relation with infertility (p=0.04). 

So, the mean±SD of sperm count, motility, and morphology in the case group was lower than controls. There was a significant relationship between these parameters and infertility. Also, there was a significant relationship between sperm count and frequency of CMV DNA in the case group (p=0.014) (Table II). In addition, a significant correlation was found between the frequency of CMV DNA and motility using Man Whitney test (p=0.025) in the case group. 

In table III, the frequency of CMV DNA is shown based on four age groups and it is higher in the age group of 35-40 yr. Finally, there was no significant relationship between age and infertility in men. No significant difference was found between age, volume of semen, and morphology in case and control groups, while mean of motility and sperm count were significantly different in positive and negative patients with CMV DNA.

**Table II:** Frequency of CMV DNA in two study groups

**Groups **		**Number (%)**	**CMV DNA**
Case group			
	Oligo spermia	39 (39)	7 (17.9)
	Azoo spermia	19 (19)	1 (5.3)
	Motility less than 40%	42 (42)	15 (35.7)
Control group		100	7 (7)
Total		200	30 (15)

Data presented as n (%); Sperm counts and motility in the control group were more than the case group (p˂0.0001)

CMV: Cytomegalovirus

**Table III:** Distribution of CMV DNA in different age groups

**Age group (yr)**	**CMV positive**	**CMV negative**
<25	9 (30%)	32 (18.8%)
25-35	9 (30%)	59 (34.7%)
35-45	10 (33.3%)	60 (35.3%)
>45	2 (6.7%)	19 (11.2%)
Total	30 (15%)	170 (85%)

Data presented as n(%); Chi-square test: p-value= 0.524

CMV: Cytomegalovirus

## Discussion

In this study, the prevalence of CMV DNA and its association with male infertility was investigated. Infertility in men is mostly without a known cause. The present study was designed to investigate the prevalence of HCMV DNA in fertile and infertile men's semen samples by using a real time PCR method. Finally, the relationship between viral presence and semen parameters was investigated. The results of this study showed a high prevalence of HCMV DNA in semen samples, in total (15%) in men referred to the IVF center, 23% in infertile men, and 7% in fertile men. 

In a study by Baghdadi and colleagues at Arak, located in West Iran, the prevalence of CMV DNA in 50 samples was analyzed. In 3 samples (6%) from infertile men and 2 samples (4%) of fertile men the virus was detected (11). Habibi and colleagues examined the prevalence of CMV virus in 154 infertile and 46 fertile of men; virus was detected in 20 samples (13%) of infertile men and 5 samples (10.86%) of the fertile men (12). In these two studies, which was done by conventional PCR method, due to insufficient number of samples, there was no significant correlation between infertility and CMV virus. However, there was a significant relationship between CMV prevalence and sperm count and motility parameters in semen sample. In a study by Wits Craig *and colleagues *at a university in Texas, which was performed by PCR, 18/72 samples (25%) was positive for CMV DNA (13). 

Also, in another study by Yang and colleagues in Taiwan, from 248 individuals referred to the laboratory for fertilization, 83 samples (33.5%) were positive for CMV DNA by Dot-blot-DNA hybridization (14). In these two studies, the high prevalence of CMV virus has been reported, due to more number of samples and the use of more sensitive methods for detecting viral nucleic acid, as well as the prevalence of endemic viruses and the inappropriateness of sexual cares and high-risk behaviors in the region (15). In present study, there was a significant relationship between infertility in men and the prevalence of the CMV infection in the semen of infertile men in Kerman. Other studies that show high prevalence of CMV infection in infertile men's semen sample, that have confirmed this relationship in our study (5, 16, 17). 

**Limitation**

Our research limitations are the small number of samples.

## Conclusion

In conclusion, the findings of this study indicate that infection with HCMV can effect on the some of essential and important factors in semen that possibly infertility in the infected men. Quick detection and on time of this viral infection by real time PCR technique will permit the suitable antiviral therapy to increase the possibility of fertility. However, this study showed a significant relationship between prevalence of CMV virus and male infertility that could be helpful considering HCMV positive cases in male infertility, as well as, removing positive cases that referring for sperm donation. 
